# Data-Driven Audiogram Classification for Mobile Audiometry

**DOI:** 10.1038/s41598-020-60898-3

**Published:** 2020-03-03

**Authors:** François Charih, Matthew Bromwich, Amy E. Mark, Renée Lefrançois, James R. Green

**Affiliations:** 1 Department of Systems and Computer Engineering, Carleton University, Ottawa, ON Canada K1S 5B6; 2 Children’s Hospital of Eastern Ontario, Ottawa, ON Canada K1H 8L1; 3 Faculty of Medicine, University of Ottawa, Ottawa, ON Canada K1H 8M5; 4 SHOEBOX Inc., Ottawa, ON Canada K3S 5R5

**Keywords:** Health care, Medical research

## Abstract

Recent mobile and automated audiometry technologies have allowed for the democratization of hearing healthcare and enables non-experts to deliver hearing tests. The problem remains that a large number of such users are not trained to interpret audiograms. In this work, we outline the development of a data-driven audiogram classification system designed specifically for the purpose of concisely describing audiograms. More specifically, we present how a training dataset was assembled and the development of the classification system leveraging supervised learning techniques. We show that three practicing audiologists had high intra- and inter-rater agreement over audiogram classification tasks pertaining to audiogram configuration, symmetry and severity. The system proposed here achieves a performance comparable to the state of the art, but is significantly more flexible. Altogether, this work lays a solid foundation for future work aiming to apply machine learning techniques to audiology for audiogram interpretation.

## Introduction

The consequences of hearing loss are frequently underestimated. The World Health Organization (WHO) has referred to the condition as a “silent epidemic”^1^. Currently, 350 million individuals live with some form of hearing loss worldwide^2^. Future projections are also grim, as this figure is expected to climb to 630 million within the next decade or so^1^. Collectively, the burden of hearing loss in the global economy is estimated at US$ 750 billion per year^3^. In children, hearing loss has been show to impede language acquisition^4–6^, and, as a result, academic performance. Adults, on the other hand, often experience feelings of isolation and depression^7^. Some studies have also shown a correlation between hearing loss and a decrease in cognitive function in older adults^8,9^. It follows that there is significant incentive to promptly identify and address hearing impairement in affected individuals.

The audiology community acknowledges a growing shortage of expertise that is not limited to developing countries, but that also affects industrialized countries^10–13^. Goulios and Patuzzi^11^ found that in order to meet the growing demand for audiological expertise, the number of audiologists would need to increase by over 50%. The gravity of this shortage is further compounded by the fact that the availability of audiologists is unevenly distributed, with most audiologists practicing in metropolitan areas^12^. As such, there exists an urgent need for adoption of technologies capable of addressing this problem.

The audiogram is the output of a standard audiometric exam, and provides a visual representation of the subject’s hearing threshold across the frequency spectrum on an inverted graph. In fact, it is simply a plot of the discrete thresholds of hearing as a function of the frequency. These plots usually contain *air conduction thresholds* where the pure tones are presented through the ear canal by means of earphones. They may also or contain *bone conduction thresholds* representing pure tones delivered by means of a vibrator typically positioned on the mastoid process. The configuration (shape of the curves), symmetry (relationship between curves), and severity (location along the y-axis) of the hearing loss hold invaluable information pertaining to the potential causes of the hearing impairement, and are critical to virtually all hearing assessements. The shape, severity and symmetry of the audiometric curves can all inform the diagnosis, as certain configurations are characteristic to certain conditions. For examples, notches around 4,000 Hz are frequently encountered in noise-induced hearing loss^14^. On the other hand a gently sloping hearing loss along the frequency spectrum is often considered a result of aging^14^. Additionally, the knowledge of air and bone conduction thresholds can differentiate between different *types* of hearing loss, i.e. sensorineural, conductive and mixed hearing loss.

Mobile audiometry, enabled by the low cost of mobile devices such as tablets and smartphones, is now widely deployed to deliver hearing tests for clinical, research, and humanitarian applications. Mobile audiometers such as the SHOEBOX Audiometry™ system (SHOEBOX Inc., Ottawa, ON) are now capable of delivering automated hearing tests and generating audiograms with limited involvement of qualified personnel. A number of studies have shown that the measurements realized with mobile audiometers rival those of conventional audiometers^15^, even for pure tone audiograms acquired in moderately noisy environments such as waiting rooms. Owing to their convenience and relative low cost when compared to conventional audiometers, these technologies are used by researchers to study the prevalence of hearing loss in underserved communities^16–18^.

Unfortunately, mobile and automated audiometry only provide a partial solution to the audiologist shortage problem. Many users, such as primary care physicians, nurses and technicians, lack the training necessary to adequately interpret or make optimal use of the wealth of clinical information comprised in audiograms. A good understanding of the audiogram combined with a tailored questionnaire, such as the Consumer Ear Disease Risk Assessment^19^, could help in reducing the burden on the healthcare system. In fact, the National Academy of Medicine concluded that an in-person medical consultation prior to hearing aid purchase may not be necessary in all cases^19^. As such, there exists a need for decision support systems that augment the interpretability of audiograms that could enable non-experts to decide whether to refer a patient to an audiologist, a hearing instrumentation specialist, or a physician, as needed.

Audiologists use a shared language to communicate with peers, but also to describe hearing loss to patients^14^. This language describes, among other, the *configuration* of the hearing loss, or the *shape* of the audiogram, the *symmetry* across ears, the *severity* of the hearing loss, and the *site of lesion*, i.e. whether the hearing loss is sensorineural, conductive or mixed. An adequate interpretation of the audiogram is key in making the best possible clinical diagnosis, and in recommending the best treatment. Not only is the process of classifying audiograms useful for audiogram classification, it can be used to study the prevalence of different types of hearing loss.

This concept of audiogram classification has been addressed previously^20–24^. In all cases, however, the rules developed to classify audiograms for the purpose of summarization were hand-crafted. These rules are inflexible, because they classify audiograms into categories without providing a measure of confidence. For example, an audiogram could appear *flat* to one audiologist, but appear to be *sloping* in a generalized fashion to another. Both descriptions could be equally appropriate; and current classification systems fail to account for this fact. Machine learning algorithms are particularly suited to such applications, because they can learn rules directly from data, and provide a confidence estimate associated with the assigned classification. To our knowledge, the studies that applied machine learning for the purpose of classifying audiograms are few and far between. One such study successfully trained three classifiers on animal audiometric data to identify the etiology underlying certain configurations of hearing loss in humans: metabolic, sensory, mixed metabolic-sensory, and age-related^25^. Another study classified auditory profiles into one of four categories based on the degree of audibility-related and non-audibility-related distortion to optimize the selection of hearing deficit compensation strategies^26^.

Here, we present a data-driven approach to audiogram classification leveraging supervised learning. The rationale for undertaking this effort was that such an algorithm could enable the interpretation of audiograms by non-experts, facilitate the grouping of audiograms for epidemiological studies on hearing loss, and act as a training tool for audiology students. This classification algorithm could also act as a first step towards developing more sophisticated algorithms capable of suggesting a differential diagnosis for the hearing loss, the adequate referral (e.g. audiologist, physician, hearing aid specialist), *etc*.

In this paper, we present the methodology employed to assemble a high quality training set for our classification algorithm. Next, we proceed to an analysis of intra- and inter-rater reliability to validate the classification schema used here and determine whether the task can reasonably be automated, following a methodology similar to that presented in^24^. Finally, we present three components of our classification engine designed to classify audiograms by configuration, severity and symmetry for the purpose of generating an intuitive summary description of the audiogram.

## Related Work

### Audiogram classification

The practice of classifying audiograms is far from new. Its importance in research was highlighted by Raymond Carhart, the father of modern audiology, as early as 1945, when he proposed one of the first standardized audiogram classification systems^27^.

A variety of classification systems have been proposed throughout the years, most of which relying on a set of rules formulated by experts^20–23^.

Margolis and Saly^24^, unsatisfied with the complexity and rigidity of Carhart’s system, devised AMCLASS™, a rule-based system specifically for the purpose of classifying audiograms generated by an automated audiometer. AMCLASS™, the current state of the art, consists of 161 rules formulated manually to maximize the classification agreement between the system and a panel of judges on annotation tasks pertaining to the audiogram configuration, severity, symmetry, and site of lesion.

### Machine learning in audiology

Machine learning is a family of data-driven techniques that learn directly from data. Supervised learning, the branch of machine learning wherein models are trained from annotated data, is becoming increasingly popular for medical applications such as diagnostics^28^, drug response prediction^29^, and prognosis prediction^30^, to name a few.

Machine learning is also being investigated for applications in audiology. Anwar and Oakes trained a logistic regression model to predict whether a patient should be prescribed in-the-ear or behind-the-ear hearing aids^31^. More recently, Bayesian active learning methods have been applied to improve the convergence and speed of the pure tone audiometry procedure. For example, Gaussian Process (GP)-based methods have been used to predict, in real-time, the amplitude of the tone that should be presented in the next query in the threshold search^32^. A related method relying on GPs has also been used to improve the detection of noise-induced hearing loss^33^. Convolutional neural networks have been used in^34^ to classify images of eardrums as normal or abnormal. Another interesting use of machine learning in audiology relates to the detection of audiograms with potential reliability issues. We have previously shown, in^35^, that Gaussian mixture models could be used to model the audiogram density landscape and detect audiogram with improbable patterns by estimating the prior probability of encountering an audiogram. While these methods have demonstrated research potential, they have yet to to be widely adopted in practice.

## Building a high quality training set

We first sought to assemble a high quality training set from which to train our audiogram classifiers. To this end, we carefully preprocessed and sampled a number of audiograms from a large public database, and consulted practicing audiologists to review the selected audiograms. Portions of the data preparation procedure have been presented previously in^36^.

### Dataset

The National Health and Nutrition Examination Survey (NHANES) is a national health survey conducted on a continuous basis in the United States^37^. A portion of the survey assesses the hearing status of subjects through pure tone audiometry. As such, the NHANES dataset contains a large collection of basic pure tone audiograms. In this work, we retrieved the audiograms acquired between 1999 and 2012, resulting a dataset of 15,498 audiograms from participants aged between 12 and 85 years (mean: 39 ± 21 years). The audiograms were obtained using a standard pure tone audiometry protocol, using a conventional audiometer with either supra-aural or insert headphones, although it is unknown which kind was used for any specific audiogram. Air conduction thresholds were measured at 7 test frequencies: 500 Hz, 1,000 Hz, 2,000 Hz, 3,000 Hz, 4,000 Hz, 6,000 Hz and 8,000 Hz, without masking in the non-test ear. Bone conduction thresholds were not recorded in the survey.

### Preprocessing and selection

In order to ensure that only valid audiograms are presented to audiologists for subsequent annotation, we only considered audiograms that met the following criteria:

1. **Complete**: we removed incomplete audiograms where at least one of the thresholds was missing;
2. **Non-trivial**: audiograms where both ears could easily be classified with the rules described in^36^ were removed;
3. **Quality**: audiograms with inter-aural gaps greater or equal to 50 dB at two or more frequencies were discarded due to the potential interaction from the non-test ear.;
4. **Hearing loss**: audiograms within normal limits, i.e. where all thresholds were below 25 dB HL, were discarded.

In order to minimize the redundancy in our training set and optimize data annotation resources, we clustered audiograms and selected a representative from every cluster. Features for clustering were derived such that the every instance represents an ear pair. Given that we relabeled the curves as *best* and *worst* ear, the sides (left or right) that generated the curves were irrelevant and did not affect clustering. This was done as to prevent the formation of additional clusters that differ only from others because of the ears that generated otherwise identical curves. We used hierarchical clustering, using the silhouette index^38^ to determine the natural number of clusters. We sampled audiograms from these cluster representatives using an iterative greedy sampling strategy where audiograms are scored based on their *uniqueness* and *anticipated prevalence among the population*. The uniqueness of an audiogram relates to the distance of the audiogram to those sampled in preceding iterations, while the anticipated prevalence relates to the size of the cluster the audiogram represents. Additional details pertaining to the clustering, sampling procedure, and features can be found in^36^.

We assembled a final dataset comprising 270 unique audiograms. Of these audiograms, some were presented twice to assess intra-rater reliability. Some audiograms, termed "trivial”, which had been eliminated from the sampling pipeline because they could easily be classified with existing rules were added back to the final dataset. The original NHANES comprises 30% such "trivial” audiograms (4,625/15,498 ears), but sampling a dataset where this proportion is preserved would be wasteful. Furthermore, it is unclear whether this ratio would be preserved in all deployment environments. For instance, the proportion of easily classified audiograms might be larger in seniors than in children, as age-related hearing loss tends to exhibit a very predictable sloping pattern. We thus ensured that the proportion of these audiograms was no larger than 10% in the dataset to be annotated. The exact composition of the final dataset is summarized in Table 1.

**Table 1:** Audiogram set composition.

Number of presentations	Non-trivial	Trivial	Total
Once	200	20	220
Twice	40	10	100
Total	280	40	320

### Rapid audiogram annotation environment

**Figure 1:**
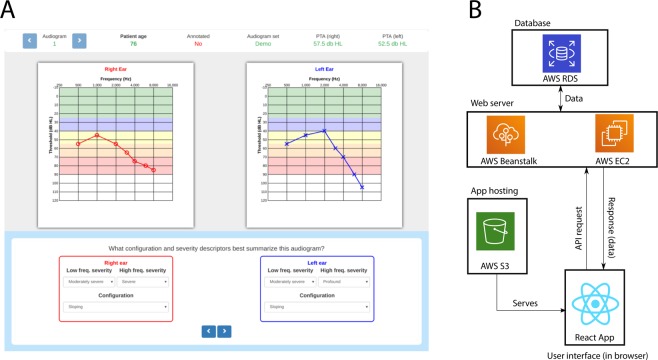
The Rapid Audiogram Annotation Environment has (**A**) an efficient user-interface and (**B**) a scalable cloud architecture.

No software was readily available to quickly and conveniently annotate large quantities of audiograms in a systematic and consistent fashion. To address this, we developed the Rapid Audiogram Annotation Environment (RAAE) shown in Fig. 1.

The RAAE was developed with modern JavaScript technologies such as React.js and Express.js to ease deployement on Amazon Web Services (AWS). An overview of the RAAE’s architecture is presented in Fig. 1B. This architecture makes the RAAE highly scalable for future use by a larger community of professionals.

In order to enforce some consistency in the collected data, the RAAE presents the audiologist with a series of questions for each audiogram. The questions are presented in Table 2. Most questions are answered by selecting the appropriate option from dropdown menus; this was done as to constrain the possible annotations.

**Table 2:** Questions posed during audiogram annotation.

Question	Possible answers
Is the audiogram symmetrical?	*Yes*, *no*, *indeterminate*
What is the configuration?^a,b^	*Flat*, *sloping*, *precipitous*, *reverse sloping*, *notched*, *cookie bite*, *reverse cookie bite*, *atypical*
How severe is the loss?^a,c^	*Within normal limits*, *mild*, *moderate*, *moderately severe*, *severe*, *profound*
Are there potentially unreliable thresholds?^a^	Possibility to click on unreliable thresholds
Are there notches?^a^	Possibility to click on thresholds in a notch

^a^On a per-ear basis. ^b^Only required for ears where there is hearing loss. ^c^The number of descriptors varies between b and c, depending on the configuration provided.

### Annotation

We organized a training session for the three licensed audiologists that were recruited to review and annotate our dataset. All of them were trained at different Canadian institutions. Two audiologists practiced in a general population, whereas the third practiced clinical audiology with pediatric patients.

The audiologists were instructed to refer to Goodman’s severity classification scale^39^ as a reference describing the severity of the hearing loss. Furthermore, the audiologists were instructed to provide a single descriptor of severity for ears with a *flat* configuration, two descriptors – for the low frequencies and high frequencies – in cases of *sloping*, *precipitous* and *reverse sloping* audiograms, and three descriptors for all remaining configurations to describe the loss in the low, mid and high frequencies. The rationale for the varying number of severity descriptors relates to the complexity of the audiogram. Much freedom was given with regards to what frequencies should be considered to belong to the low, mid, or high frequencies. Audiologists were instructed to assign the *notched* configuration to an audiogram if the audiogram has a globally normal configuration except for an audiometric notch; otherwise they should assign another descriptor corresponding to the overall configuration if a notch occurred in an audiogram. No other instructions were provided to the participants, and the number or length of sittings were left to their discretion.

Each of the three audiologists completed their annotations through the RAAE. The audiologists were shown the same 320 audiograms, but in varying orders generated randomly. We ensured that no duplicates would be shown consecutively, as this might artificially inflate intra-rater reliability.

## Rater reliability analysis

In order to train a learning algorithm on a dataset where the target variables are subjective, there should be at least *some* agreement in terms of how to describe hearing loss. To determine whether automated classification is a reasonable goal, we assessed the intra- and inter-rater reliability of the audiologists over the five annotation tasks. More specifically, we computed Fleiss’ kappa (*κ*) statistic^40^ over all five annotation tasks. This statistic is a generalization of Cohen’s kappa statistic^41^ for more than two raters, and accounts for the probability that two raters agree by chance. We used Landis and Koch’s guidelines^42^ to interpret the kappa values. All kappa calculations were conducted with the *raters* package for R^43^.

### Intra-rater reliability

**Figure 2:**
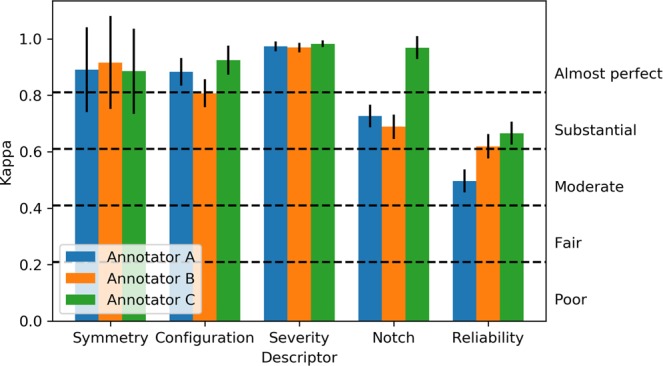
Intra-rater reliability calculated from 50 audiogram replicates (error bars represent the standard error from the mean).

We found that intra-rater reliability, measured over the repeated presentations of duplicated audiograms (see Table 1), was *moderate* or better across all tasks (Fig. 2), meaning that all three audiologists were mostly self-consistent. Intra-rater reliability was highest for description of severity, symmetry and configuration, where agreement was, on average, *almost perfect*. Agreement was somewhat lower, i.e. *substantial*, for identifying audiometric notches, and only *moderate* for identifying potentially unreliable thresholds.

### Inter-rater reliability

**Figure 3:**
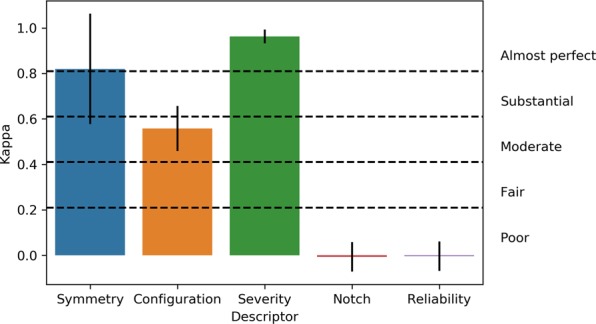
Inter-rater reliability between three professional audiologists for 270 audiograms (error bars represent the standard error from the mean).

The inter-rater reliability measures the agreement between different audiologists. This number offers insight into the extent to which training and field of practice can influence the interpretation of an audiogram. Our findings, summarized in Fig. 3, show that agreement on the severity of an audiogram was almost perfect (*κ* = 0.96 ± 0.03), which is hardly suprising given that specific directives were given regarding the scale to be used when describing severity. The lack of a precise definition as to which thresholds belong to what range, e.g. which frequencies are considered to be high frequency, may account for the minimal disagreement. Agreement was slightly lower, albeit still *almost perfect*, regarding how to best describe the symmetry of the hearing loss (*κ* = 0.84 ± 0.24). Agreement on how to best describe the configuration of the hearing loss was *moderate* (*κ* = 0.55 ± 0.10), an unsurprising finding given that this task is intuitively more complex. We found that there is essentially no agreement between the audiologists on which thresholds belong to an audiometric notch (*κ* = 0.00 ± 0.06) and which thresholds may have reliability issues (*κ* = 0.00 ± 0.06). The lack of agreement regarding the reliability of thresholds is not unexpected, given that audiologists typically leverage additional sources of information, such as the patient history or otoscopic findings, to make this determination. However, the lack of agreement with respect to the identification of thresholds located in an audiometric notch is more puzzling, suggesting that the required depth of a notch, relative to the remaining audiogram, may not be a uniformly defined concept.

Altogether, our measurements of inter-rater reliability align well with those obtained by Margolis and Saly^24^. In their experiment, they had observed slightly better inter-rater agreement on classification of configuration, but slightly worse agreement over classification of severity. These differences in agreement levels may be attributed to differences in configuration classification systems, where our scheme includes 8 configurations instead of 6 used in^24^.

We found that inter-rater reliability on the audiogram configuration classification task was lower (*p* < 0.05; unpaired Student’s *t*-test) on "challenging” ears that could not be classified with existing rules^14^ (410 ears; *κ* = 0.57 ± 0.08) than for "trivial” cases (130 ears; *κ* = 0.72 ± 0.06).

Although the agreement was better on "trivial” cases, the agreement was lower than expected. In general, these audiograms largely follow a linear trend, and as such, the configuration is assigned based on the slope of the line of best fit. However, the slope is rarely precisely measured in practice, resulting in blurred boundaries between what constitutes a *flat*, *sloping* or *precipitous* audiogram in practice. Furthermore, the visual presentation of the audiogram can drastically bias the perception of the slope. For example, an audiogram plotted such that y-axis ranges from  −10 dB HL to 120 dB HL may differ in visual appearance from one where the upper testing limit is 100 dB HL.

## Audiogram Classification

The results above indicate that while it may be possible to automate the classification of configuration, severity and symmetry, identification of threshold of questionnable reliability and audiometric notches may not be achievable consistently. For this reason, we limited the scope of our classification system, the Data-Driven Annotation Engine (DDAE), to the classification of audiograms in terms of configuration, symmetry and severity.

The DDAE was trained using the training set assembled earlier to predict the correct label(s) for each of these three descriptors. For audiograms annotated twice for intra-rater reliability estimation, we used only the second annotation. The rationale for this decision is that audiologists reported that their annotations improved throughout the annotation process, as a result of seeing a wider variety of audiograms.

### Design constraints

To improve upon existing systems, we sought to incorporate the following properties in our approach:

1. **Dimension-independence**: The system can accept a variable number of thresholds. This is enabled by the use of dimension-independent features. This property is important as many audiologists consider certain inter-octave frequencies, such as 3,000 Hz and 6,000 Hz, to be optional, leading to incomplete audiograms in certain cases. As such, the number of measurements varies from one audiogram to the next.
2. **Confidence estimates**: Provides an estimate of the confidence of the labels assigned to the audiograms.
3. **Online learning**: Can easily be retrained as new data become available.
4. **Multi-label classification (configuration)**: Relieves the assumption that a single configuration descriptor can describe the audiogram.
5. **Data-driven**: The classification rules are obtained through the optimization of objective classification accuracy criteria, instead of manually, to prevent deviations from the original expert raters of the training data.

### Problem formulation

To avoid combinatoric expansion of our problem, the questions of symmetry, severity, and configuration were addressed separately and sequentially. This was important, given that we had detailed annotations for only 270 audiograms from three expert audiologists for both training and evaluating our system. The DDAE is therefore composed of three distinct modules, each responsible for one of the following tasks:

1. **Configuration labelling**: Classification of ears by configuration using an ensemble of decision trees, which we term *decision forest* – not to be confused with the widely known *random forest* classifier. Every tree in the forest is a binary classifier corresponding to one configuration, whose goal is to determine whether the corresponding configuration accurately describes the audiogram, partially or in its entirety. In this formulation, configurations are not mutually exclusive.
2. **Severity labelling**: Assignment of 1, 2 or 3 severity labels to an ear, depending on the predicted configuration(s).
3. **Symmetry labelling**: Binary classification problem determining whether the audiogram is symmetrical or asymmetrical.

### Classification of configuration

**Figure 4:**
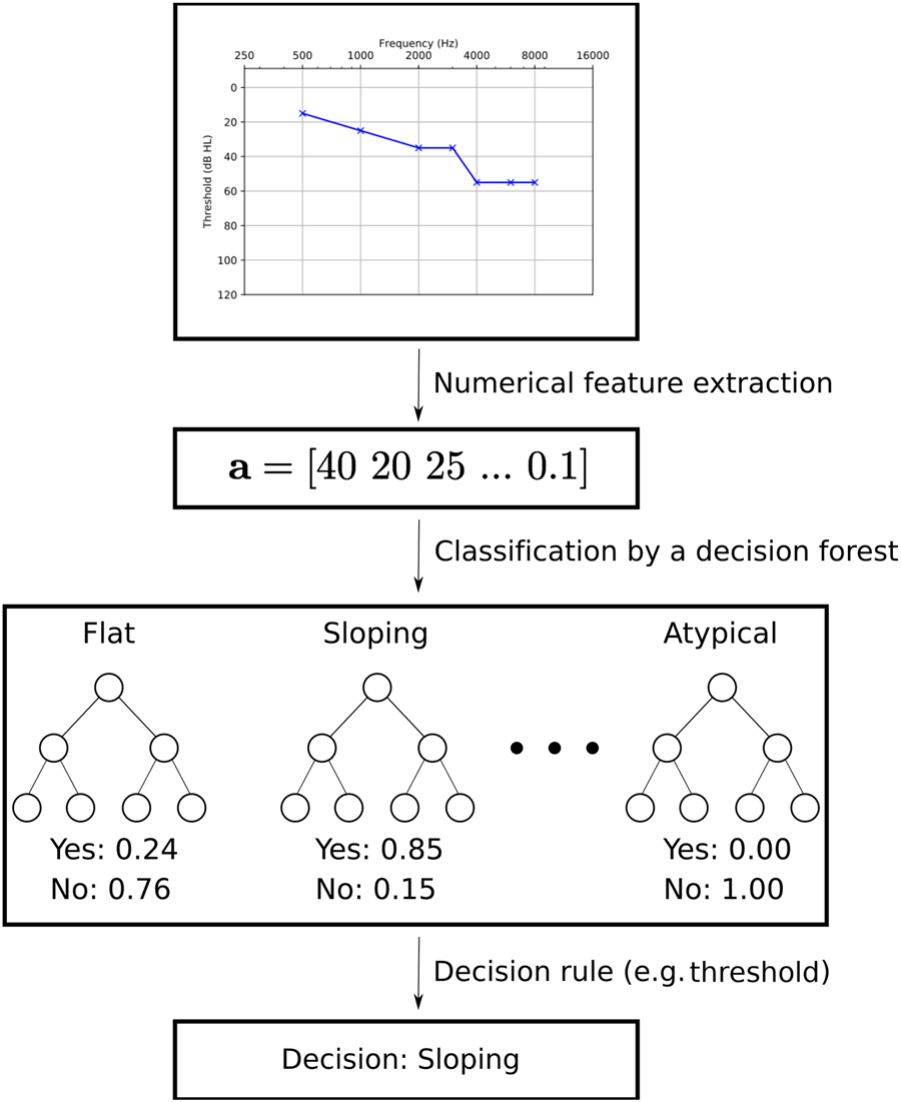
Configuration classification decision forest.

The design of the audiogram configuration classification pipeline is illustrated in Fig. 4.

We derived a total of 15 features (Table 3) from the thresholds of each audiogram. To our knowledge, these features had not been used previously by other groups. These features have the benefit of remaining interpretable in addition to allowing the classifier to handle a varying size input. Given the relatively small size of the training set, the depth of each decision tree was limited to prevent overfitting^45^. Therefore, only a subset of the features in Table 3 were incorporated into the actual decision forest, selected by the entropy-based feature selection inherent in the decision tree training process.

**Table 3:** Features defined for the configuration classification models.

	Description
1	Slope of the line of best fit
2	Proportion of positive slopes joining consecutive thresholds
3	Proportion of negative slopes joining consecutive thresholds
4	Maximum threshold (worst threshold)
5	Minimum threshold (best threshold)
6	Average threshold
7	Standard deviation of the thresholds
8	Average of thresholds in the low frequency range (below 1,000 Hz)
9	Average of thresholds in the mid frequency range (between 1,000 Hz and 3,000 Hz)
10	Average of thresholds in the high frequency range (4,000 Hz and above)
11	Proportion of slopes that change signs with respect to the previous slope
12	Mean absolute residual from the line of best fit
13	Audiogram curvature; highest-order coefficient of the quadratic of best fit
14	Audiogram range; difference between the maximum and minimum thresholds
15	Notch index^44^

Using the scikit-learn optimized implementation of the CART decision tree learning algorithm^46^ and information gain as the quality criterion, we trained the decision trees using 3-fold class-stratified cross-validation. For each configuration, we assumed the ground truth to be positive if one of the three audiologists had assigned that particular configuration to the audiogram. This decision reflects the realization that an audiogram may be equally well described by multiple configuration labels. We varied hyperparameters controlling the depth of the tree, and selected, for all trees, the model that maximized the average *F*_1_ score over the three folds. The maximal allowable depth was 5, to limit the odds of overfitting and for facilitating the interpretation of the resulting rules. Finally, we trained the model on the entire dataset using the optimal tree depth. Given the small dataset size, all data were used for training and the performance metrics presented here represent the mean value over the three folds for the optimal set of hyperparameters.

For each decision tree output, confidence was measured as the fraction of the training samples from the majority class in the leaf node corresponding to the test instance^46^. In other words, the purity, or the fraction of the majority class of the leaf node, was taken to be the confidence estimate.

The performance of every individual tree in the forest in 3-fold cross-validation is shown in Table 4, where it is compared with AMCLASS™ whose performance was assessed through bootstrap sampling with 1000 bootstrap samples of size 270. The rationale for using 3-fold cross-validation for this task is that there is a high class imbalance in the labels, and splitting in 5 or 10 folds may cause certain folds to contain no instances of rare classes (e.g. *cookie bite*). Given that AMCLASS™ uses a different configuration classification scheme, we established an equivalence scheme, and considered the predictions made by AMCLASS™ to be correct if they were equivalent to one of the labels provided by one of our three audiologists. For example, a “trough-shaped” audiogram was considered to be equivalent to a *cookie bite* configuration. A *sloping* prediction in AMCLASS™ was considered to correct if our annotation indicated a *sloping* or *precipitous* configuration, as no distinction between the *sloping* and *precipitous* configurations is made in AMCLASS™.

**Table 4:** Performance of our configuration classifiers.

	DDAE				AMCLASS™			
	Accuracy	Recall	Precision	*F*_1_	Accuracy	Recall	Precision	*F*_1_
Flat	0.94 ± 0.02	0.84 ± 0.10	0.80 ± 0.05	0.81 ± 0.07	0.90 ± 0.02	0.66 ± 0.10	0.47 ± 0.09	0.55 ± 0.08
Sloping	0.81 ± 0.07	0.81 ± 0.04	0.81 ± 0.04	0.81 ± 0.04	0.81 ± 0.02	0.80 ± 0.03	0.88 ± 0.03	0.84 ± 0.02
Precipitous	0.88 ± 0.01	0.82 ± 0.03	0.85 ± 0.02	0.83 ± 0.01				
Reverse sloping	0.96 ± 0.01	0.83 ± 0.06	0.81 ± 0.05	0.81 ± 0.01	0.81 ± 0.02	0.80 ± 0.03	0.88 ± 0.03	0.84 ± 0.02
Cookie bite	0.95 ± 0.01	0.62 ± 0.02	0.74 ± 0.06	0.65 ± 0.01	0.96 ± 0.01	0.38 ± 0.14	0.63 ± 0.19	0.46 ± 0.14
Reverse cookie bite	0.93 ± 0.01	0.80 ± 0.06	0.83 ± 0.05	0.81 ± 0.04	0.93 ± 0.02	0.66 ± 0.09	0.66 ± 0.09	0.66 ± 0.08
Notched	0.84 ± 0.04	0.71 ± 0.04	0.70 ± 0.06	0.70 ± 0.05	N/A	N/A	N/A	N/A
Atypical	0.84 ± 0.04	0.61 ± 0.03	0.70 ± 0.13	0.62 ± 0.04	0.75 ± 0.03	0.09 ± 0.03	0.61 ± 0.16	0.15 ± 0.05

The DDAE’s performance is, in general, on par with that of AMCLASS™, performing slightly better for certain categories and slightly worse on others. The difficulty of our classification problem was harder, however, as our configuration scheme was more fine-grained, as discussed previously. The low performance of the tree responsible for the *atypical* configuration aligns well with the notion that an *atypical* audiogram is not a single concept, but rather multiple concepts. As such, the DAAE only predicts an *atypical* configuration when none of the other configuration’s confidence estimate meets a cut-off threshold to be set by the system’s user, e.g. 50%. Interestingly, one may notice the lower accuracy achieved over the *sloping* class than over the *atypical* class. While surprising at first glance, we suspect that this may be a result of the vagueness of the boundaries separating *sloping* audiograms from *flat* or *precipitous* ones. In other words, *sloping* audiograms are easily misclassified with closely related configurations. In contrast, the *atypical* configuration, our catch-all configuration, is very broad and very different from other configurations.

### Classification of severity

Normally, to quantify severity of hearing loss, one would first compute a pure tone average, and look in a reference table for the correct descriptor (e.g. *mild*) corresponding the decibel value. In reality, a single severity descriptor is only sufficient in *flat* hearing losses. For *sloping*, *precipitous* and *reverse sloping* audiograms, two descriptors are required to describe the severity in the best and worst ranges (low frequencies and high frequencies, or vice-versa) across the frequency spectrum. For *cookie bite*, *reverse cookie bite*, *notched* and *atypical* audiograms, it is very difficult to accurately convey the extent of the loss across the spectrum with fewer than three descriptors. As such, this module in the DDAE predicts one or more severity labels for each configuration assigned to the audiogram by the previous module.

Goodman defined a severity scale in the form of a table^39^ which is a mapping *g* of the form:

1 $$g:t\to l$$

where *t* ∈ [−10, 120] is an audiometric threshold and *l* ∈ *L* is one of the possible severity labels, with the set of all possible labels defined below:

2 $$L=\{normal,\,mild,\,moderate,\,moderately\,severe,\,severe,\,profound\}$$

The goal of the DDAE severity classifier “training” procedure was to find, given configuration *c*, and a frequency range *i*, the optimal feature *f* ^*^. In this context, features can be thought of as functions that map an audiogram *a* to a real number:

3 $$f:a\to {\mathbb{R}}$$

The set of possible features is presented in Table 5 and borrows from features defined in other works^24^. The optimal feature was defined to be the one which most often predicted the label assigned by the audiologists when mapped to a label with *g*:

4 $${f}_{c,i}^{* }={{\rm{argmax}}}_{f}{\sum }_{r\in R}{\sum }_{a\in {A}_{r,c}}[g(f(a))={l}_{a,i}]$$

**Table 5:** Features defined for severity classification.

	Description
1	Average threshold
2	Maximum (worst) threshold
3	Minimum (best) threshold
4	Average of thresholds in the low range
5	Maximum (worst) threshold in the low range
6	Minimum (best) threshold in the low range
7	Average of thresholds in the mid range
8	Maximum (worst) threshold in the mid range
9	Minimum (best) threshold in the mid range
10	Average of thresholds in the high range
11	Maximum (worst) threshold in the high range
12	Minimum (best) threshold in the low range
13	Maximum (worst) threshold in notch-susceptible frequencies (between 3,000 and 6,000 Hz, inclusively)

Low range is defined as frequencies below 1,000 Hz, the mid range includes frequencies between 1,000 Hz and 3,000 Hz inclusively, while the high range comprises all frequencies greater or equal to 4,000 Hz.

where *r* is one of the three audiologists, *A*_*r*,*c*_ is the set of audiograms annotated by *r* that have configuration *c*, and *l*_*a*,*i*_ is the true label selected to describe the severity of the hearing loss in the frequency range *i* of audiogram *a*. The Kronecker delta function in Iverson notation, [⋅], equals 1 if the argument is true, and 0 otherwise.

Less formally, one can say that the “training” or “feature selection” procedure for the severity classification system consists of identifying the feature that most often produces the label provided by the audiologists when the feature value is looked up in Goodman’s severity scale^39^. This feature selection is repeated for all configurations, and for all relevant frequency ranges associated with the configurations.

  Table 6 presents the accuracy of our predictor over all configurations and their associated frequency ranges of interest. The accuracy of our predictor is generally very good, as seen in 5-fold cross-validation. Accuracy was lowest for prediction of the severity of audiograms that had been classified as *notched* or *atypical*.

**Table 6:** Accuracy of the severity prediction module of the DDAE.

Configuration	Lows	Mids	Highs
Flat	0.87 ± 0.13	N/A	N/A
Sloping	0.93 ± 0.02	N/A	0.96 ± 0.01
Precipitous	0.95 ± 0.03	N/A	0.97 ± 0.02
Reverse sloping	0.98 ± 0.05	N/A	0.97 ± 0.05
Cookie bite	0.98 ± 0.04	1.00 ± 0.00	0.97 ± 0.05
Reverse cookie bite	0.91 ± 0.09	0.99 ± 0.03	0.93 ± 0.06
Notched	0.98 ± 0.02	0.72 ± 0.02*	0.54 ± 0.17
Atypical	0.81 ± 0.16	0.66 ± 0.30	0.71 ± 0.13

*This descriptor represents frequencies most susceptible to host audiometric notches (i.e. 3,000 Hz, 4,000 Hz and 6,000 Hz), and corresponds to the deepest threshold in the notch.

### Classification of audiogram symmetry

Classification of symmetry is a binary problem requiring a single decision tree instead of a forest, given that there were three audiologists and only two possible labels.

Following the work of Margolis *et al*.^24^, we considered a set of 6 features listed in Table 7. Where a threshold was only available for one ear, the threshold for the other ear was interpolated linearly using neighbouring thresholds. If this occurred at the lowest or highest frequency, we chose to eliminate the threshold from the features to avoid extrapolation.

**Table 7:** Features defined for the symmetry classification model.

	Description
1	Maximum inter-aural threshold difference
2	Minimum inter-aural threshold difference
3	Average inter-aural threshold difference
4	Average inter-aural threshold difference
5	Difference in the slopes of the lines of best fit
6	Difference between the average threshold across ears

Results from 5-fold cross-validation testing indicate that our decision tree trained to classify audiograms by symmetry performs slightly better than the widely applied classification rule described in^24^ that counts interaural threshold differences greater or equal to 20 dB on our dataset (Table 8). In fact, our method achieves a better *F*_1_ score, although statistical significance could not be achieved (*p* = 0.83).

**Table 8:** Performance on symmetry classification.

Configuration	Our method	Existing rule
Accuracy	0.98 ± 0.02	0.89 ± 0.02
Recall	0.94 ± 0.05	1.00 ± 0.00
Precision	0.99 ± 0.01	0.88 ± 0.02
*F*_1_	0.96 ± 0.04	0.93 ± 0.01

## Conclusion

In this paper we presented the systematic development of a fully data-driven audiogram classification system.

First, we presented a strategy to select the most informative audiograms from a large database of audiometric data. Using the RAAE, a web-based annotation software built specifically for this study, we collected 320 audiogram annotations from three licensed audiologists.

Next, we showed that intra-rater reliability for the classification of audiogram configuration, symmetry and severity range from *moderate* to *almost perfect*. This agreement was maintained, albeit at a lower level, when considering agreement between audiologists. There was no agreement with respect to what constitutes an audiometric notch and which thresholds may suffer from data quality issues. This suggested that there is sufficient agreement between audiologists for classification of configuration, severity and symmetry for these classifications to be automated.

Finally, we presented the DDAE, a system consisting of three separate machine learning modules designed to classify audiograms by configuration, symmetry and severity. Our system achieved a performance comparable to the state of the art on our dataset in cross-validation in terms of classifying audiogram configuration and symmetry.

Our approach is significantly more flexible than the existing classification systems. First, in contrast to existing methods, the DDAE achieves data-driven decision logic rather than relying on expert-tuned rule sets. This makes it a much more flexible approach. This flexibility enables the derivation of rule sets that are specific to sub-populations of interest, for applications such as workplace monitoring or pediatric care. Second, our systematic framework for developing a classification system is amenable to online learning, where newly acquired and labelled audiograms can be added to the training data to further refine the decision logic. With wide deployment of mobile audiometry systems, such as SHOEBOX, thousands of new audiograms can be collected monthly, many of which will have been annotated by expert audiologists. These “high confidence” annotated data could be incorporated into the training set, by integrating the RAAE within mobile audiology platforms such as SHOEBOX. Finally, the RAAE presented here can be used to train audiologists, and even non-experts. Manually entered classifications can be compared with expert consensus. It may be possible to apply the DDAE to generate expert-level classifications for the large body of unlabelled audiograms available to further augment the training sets available to students of audiology. For this purpose, synthetic data could also be generated and labelled using the DDAE. Additional advantages of our approach include confidence estimates for classifications of configuration and symmetry, the possibility to assign multiple configurations to an audiogram, and the use of dimension-independent features that enable the system to classify audiograms with varying numbers of thresholds.

It may be unclear why we elected to treat the classification of configuration as a multi-label problem. This is explained by two reasons, one fundamental in nature, and the other logistical in nature. The first one relates to the fact that in some cases, as mentioned previously, more than one descriptor of configuration may accurately describe the hearing loss. The second one is that using a strategy where the algorithm is trained on audiograms for which a consensus was achieved may lead to reduction in the number of audiograms available for training. This is particularly undesirable in small datasets. For the task of configuration classification, a consensus was achieved for only 310/540 ears (57.4%). Another approach to mitigate this would be to use a majority voting approach to obtain a single configuration label for the audiogram. This approach however still leads to loss of valuable data, more specifically 43/540 ears (8.0%).

Non-expert users of mobile and automated audiometry devices will benefit most from this audiogram classification system which can empower them to make better decisions when faced with certain types of audiograms. The benefits of clinical decision support systems such as the one presented here have been demonstrated in many fields, including ECG interpretation^47^ for telemedicine for example. Furthermore, the system presented here will enable expert audiologists to devote more time engaging with the patient regarding their condition, as annotations will be automatically generated.

Of course, certain limitations are associated with the work presented here one of which relates to the size of the dataset used to train the DDAE. Due to the logistical complexity and cost of acquiring audiogram annotations, we were only able to assemble a dataset of 270 distinct audiograms annotated by 3 separate audiologists. While we did ensure that our audiologists were trained in different schools of audiology and practiced audiology with different subpopulations, it is likely that our estimate of inter-rater reliability could be made more accurate by adding additional raters. In fact, hiring more audiologists and collecting more audiograms would likely further increase our confidence that these results can be generalized. Specifically, adding more raters is likely to increase inter-rater reliability (but not intra-rater reliability, which is reflection of the inherent difficulty of the task). Unfortunately, augmenting our dataset is extremely costly, as the professional services of multiple audiologists are required. If large public datasets, such as the NHANES, were to include diagnostic outcome, then this would enable larger scale studies in the future. A second major limitation worth mentioning is that the classification system presented here cannot classify audiograms by site of lesion, while AMCLASS™ can. Obtaining labels for this descriptor of hearing loss was impossible because the unlabeled NHANES data used in this study did not contain masked or unmasked bone conduction thresholds. Finally, while a step in the right direction, the NHANES dataset used in this study did not comprise the data necessary to extend our algorithm such that it can identify a potential diagnosis or the appropriate professional to whom the patient should be referred.

Future work will aim to collect more data and to investigate the integration of additional sources of data such as medical history, patient age, bone conduction thresholds, questionnaire data, otoscopic images, and tympanogram data. The ultimate goal is to extend the scope of this system, such that it not only describes the audiogram, but also provides a proposed differential diagnosis. Additionally, the system could eventually provide recommendations with respect to referral and treatment options. Another avenue involves assessing the generalizability of our system, although this will involve labeling additional audiograms to validate the DDAE against. Finally, when undertaking this project, we sought to examine whether machine learning can accomplish the same audiogram classification tasks normally completed by a professional audiologist. Future studies should examine additional novel applications of machine learning in the field of audiology, beyond automating the state of the art. However, adoption of such innovations may require a change in the practice of audiology itself and are beyond the scope of our present study.

Taken together, this work makes a strong case for the use of machine learning for audiogram interpretation and provides a data annotation and classification framework to support such endeavours.

## Data Availability

The NHANES dataset can be retrieved online (https://www.cdc.gov/nchs/nhanes/index.htm). The anonymized audiogram annotation data will be provided upon request.
